# Invasive infection caused by *Klebsiella pneumoniae* is a disease affecting patients with high comorbidity and associated with high long-term mortality

**DOI:** 10.1371/journal.pone.0195258

**Published:** 2018-04-06

**Authors:** M. Vading, P. Nauclér, M. Kalin, C. G. Giske

**Affiliations:** 1 Division of Clinical Microbiology, Department of Laboratory Medicine, Karolinska Institutet, Stockholm, Sweden; 2 Department of Infectious Diseases, Danderyds Hospital, Stockholm, Sweden; 3 Department of Infectious Diseases, Karolinska University Hospital Solna, Stockholm, Sweden; 4 Department of Medicine Solna, Infectious Diseases Unit, Karolinska Institutet, Stockholm, Sweden; 5 Department of Clinical Microbiology, Karolinska University Hospital Solna, Stockholm, Sweden; Cornell University, UNITED STATES

## Abstract

*Klebsiella pneumoniae* (KP) is after *Escherichia coli* (EC) the most common gram-negative species causing invasive infections. Herein, we analyzed risk factors and prognosis in invasive infections caused by KP versus EC, in an area with low antimicrobial resistance. Moreover, we compared antimicrobial resistance and relative prevalence of KP and EC (KP/EC-ratio) in different European countries, using EARS-Net data. Adult patients admitted to Karolinska University Hospital 2006–2012 with invasive infection caused by KP (n = 599) were matched regarding sex and age with patients infected by EC. The medical records were retrospectively reviewed. Comorbidity was adjusted for with multivariable analysis. European data were retrieved from the EARS-Net database. No differences were observed in 7- and 30-day mortality between the groups. The 90-day mortality was significantly higher in the KP cohort (26% versus 17%, p<0.001), but not after adjusting for comorbidity. Malignancy was seen in 53% of the patients with KP versus 38% with EC, OR 1.86 (1.34–2.58). A significant increase in the rate of ESBL-production was observed in EC, but not in KP. The KP/EC-ratio remained stable. In contrast, European data showed increasing percentages of isolates non-susceptible to third-generation cephalosporins in EC and KP, and increasing KP/EC-ratio. Invasive infection caused by KP is a disease affecting patients with high comorbidity and associated with high 90-d mortality. The stable KP/EC-ratio and low occurrence of antimicrobial resistance in data from Karolinska University Hospital compared to aggregate data from 20 EARS-Net countries could be related to absence of clonal spread of multidrug-resistant KP.

## Introduction

*Klebsiella pneumoniae* is second to *Escherichia coli* the most common gram-negative pathogen associated with a wide spectrum of infections, such as urinary tract infection (UTI), pneumonia, intra-abdominal infection, bloodstream infection (BSI), meningitis and pyogenic liver abscess (PLA) [1–4]. During the last decades the rates of extended-spectrum cephalosporin-resistant *K*. *pneumoniae* producing extended-spectrum β-lactamases (ESBL) have dramatically increased worldwide, and in most parts of the world *K*. *pneumoniae* is the pathogen mostly associated with dissemination of ESBLs and other horizontally transmissible resistance genes [5, 6].

Invasive infections caused by *K*. *pneumoniae* have been associated with comorbidities such as cancer, diabetes, and previous organ transplantation [7, 8]. A high case fatality rate has been reported, ranging between 18 and 49%, where more recent studies focus on infections caused by multi-drug resistant isolates [3, 7, 9–13]. A population-based study on bloodstream infection (BSI) caused by *K*. *pneumoniae* 2000–2007 in Canada, a setting with low prevalence of antimicrobial resistance, showed an increase of the burden of disease during the last decade and a case fatality rate of 19% [7]. In the same demographic area 2000–2006 the case fatality rate in BSI caused by *E*. *coli* was 11% [14]. Studies comparing community- and hospital-acquired BSI caused by *K*. *pneumoniae* demonstrate differences in risk factors and outcome [10, 15, 16], where neoplastic disease and antimicrobial resistance are common among patients with hospital-acquired infections. In a few studies BSI caused by *K*. *pneumoniae* or *E*. *coli* have been compared but there is still limited data on early and late mortality and differences in risk factors for acquisition between patient groups affected by the respective pathogens [11, 17].

The primary aim of this study was to compare risk factors for acquisition and mortality, using the endpoints 7-, 30-, and 90-day mortality, of invasive infection caused by *K*. *pneumoniae* compared to *E*. *coli*. A secondary aim was to use European EARS-Net data on invasive infections to compare temporal trends in rates of resistance in *E*. *coli* and *K*. *pneumoniae* and relative occurrence of the two species, pursuing the hypothesis that some resistant clones of *K*. *pneumoniae* may contribute to a species-shift, as measured by the ratio between K. *pneumoniae* and *E*. *coli* (KP/EC-ratio).

## Methods

### Study populations and bacterial isolates

#### Data from Karolinska University Hospital, Sweden

All adult (≥18-year-old) patients admitted to Karolinska University Hospital, Sweden, a tertiary medical center with 1700 beds, between 2006 and 2012, with growth of *K*. *pneumoniae* either in blood (n = 646), cerebrospinal fluid (CSF) (n = 4), or both (n = 2) were included in this retrospective cohort study (S1 Fig). These patients are referred to as the “extended *K*. *pneumoniae* cohort”. Isolates and patients were identified by searches in the clinical microbiology laboratory information system at the Karolinska University Laboratory. Species identification was done with the API 20E system (bioMérieux, Marcy l’Etoile, France) or VITEK2 (bioMérieux). Antimicrobial susceptibility testing was performed with the disk diffusion method on Isosensitest agar (Oxoid, Basingstoke, UK) and interpreted according to the guidelines of the Swedish Reference Group for Antibiotics (SRGA) [18]. Co-infection with *E*. *coli* was seen in 53/652 patients (8%). The 599 patients without co-infection with *E*. *coli* are referred to as the “*K*. *pneumoniae* cohort”. For each patient in the *K*. *pneumoniae* cohort a patient with invasive (BSI or meningitis) *E*. *coli* infection, matched 1:1 regarding sex, age and year of onset of infection, was selected at random from patients in the microbiological database. These 599 patients are referred to as the “*E*. *coli* cohort”. For all study subjects (n = 1,251) the medical records were reviewed by an infectious disease specialist regarding patient risk factors, hospital- versus community-acquired disease, source of infection, antimicrobial treatment, and mortality. Only the first episode of an infection during the study period was included in the analyses.

### Definitions

In this study *K*. *pneumoniae* refers to *K*. *pneumoniae* sensu latu [19]. Infections were defined as hospital-acquired if the sample was obtained >48 h after admittance. Healthcare-associated community-onset infection was defined as either having been admitted to hospital, having had surgery, received outpatient hemodialysis, living at a long-term care facility, or attended daycare within the previous 30 days before the onset of infection [20, 21]. For comparison of antimicrobial resistance with EARS-Net data the same algorithm as for international data (i.e. one isolate per year and patient, and inclusion of all ages) was used. The infection was classified as polymicrobial if at least one additional species was recovered from blood specimens drawn within 24 h from the recovery of *K*. *pneumoniae*. Presence in one blood culture bottle was considered sufficient except for frequent skin contaminants that had to be present in at least two venipunctures [22]. Comorbidities were analyzed individually and as Charlson comorbidity index [23].Time to antimicrobial therapy was defined as time from arrival to hospital or, when onset at hospital, time from onset of symptoms until administration of adequate antimicrobial therapy. Adequate antimicrobial therapy was defined as treatment including at least one antibiotic susceptible to the present pathogen/pathogens. Data on combination versus single therapy was not evaluated.

#### Data from the European Antimicrobial Resistance Surveillance Network (EARS-Net)

The European Antimicrobial Resistance Surveillance Network (EARS-Net) is an international surveillance network that collects routine clinical antibiotic susceptibility data from all 28 European Union (EU) Member States and two European Economic Area (EEA) countries, Iceland and Norway. The network is coordinated by the European Centre for Disease Prevention and Control (ECDC). Only invasive isolates from blood or cerebrospinal fluid are included in the EARS-Net data. The AST results are ascertained according to agreed protocols [24, 25], and the general quality and comparability of the data are evaluated through an annual external quality assessment offered to the participating laboratories.

Data on invasive *E*. *coli* and *K*. *pneumoniae* isolates with AST information for third-generation cephalosporins reported for the period 2006–2012 were extracted from the database at ECDC. Resistance to third-generation cephalosporins was defined as resistance to at least one of the third-generation cephalosporins under surveillance by EARS-Net: ceftriaxone, ceftazidime or cefotaxime. Isolates were considered as non-susceptible for this study when tested and interpreted as intermediate (I) or resistant (R) in agreement with the clinical breakpoint criteria used by the local laboratory. To reduce sampling bias, countries either reporting few isolates (median value of <300 *E*. *coli* isolates per year), or lacking data for parts of the period, were excluded. Twenty countries were included in the analysis: Austria, Czech Republic, Germany, Denmark, Greece, Finland, France, Hungary, Ireland, Italy, Lithuania, Luxemburg, the Netherlands, Norway, Poland, Portugal, Spain, Sweden, Slovenia, and the United Kingdom.

### Statistical analysis

Sample size was based on calculation of a 6% difference in mortality within 30 days (17 and 11% respectively for the *K*. *pneumoniae* and *E*. *coli* cohort) with a power of 0.8. Conditional logistic regression was used when comparing variables (clinical characteristics) between the *K*. *pneumoniae* cohort and the *E*. *coli* cohort. For categorical variables within the cohorts, Fisher´s exact test and Chi-square test were used. The Mann Whitney test was used to compare continuous variables. For all tests a two-sided p-value <0.05 was considered significant. A multivariable model was built based on stepwise forward and backward selection using the parameters with a p-value <0.2 in univariate analysis and comparing models with the likelihood ratio test (LRT). Of the strongly correlating risk factors only one was picked. Odds Ratio (OR) and 95% Confidence Intervals (CI) were calculated. Since hospital-acquired infection might correlate with other comorbidities we also performed a model without including the variables hospital-acquired, healthcare-associated community-onset and community-acquired infection. These results (data not shown) were similar to the results of our final model. To analyze data on mortality logistic regression was used, and similar multivariable models were built, restricted by the number of outcomes.

For international comparison, *K*. *pneumoniae* to *E*. *coli* ratios and percentages of *K*. *pneumoniae* and *E*. *coli* isolates non-susceptible to third-generation cephalosporins were calculated for each data source (Karolinska University Hospital and EARS-Net), country and year. The association between the national KP/EC ratio and the percentage third-generation cephalosporins non-susceptible isolates was assessed by Pearson correlation coefficient. The command ptrend in Stata was used for trend analysis of proportions. For EARS-Net data, a population-weighted EU/EEA mean resistance percentage was determined by applying population-based weights to each country’s data before calculating the arithmetic mean for all reporting countries. Country weights were used to adjust for imbalances in reporting propensity and population coverage, as the total number of reported isolates per country in most cases does not reflect the population size. The weight applied to each national data point represented the proportion of the country’s population out of the total population of all countries included in the calculation. Annual population data were retrieved from the Eurostat on-line database [26].

All statistical analyses were performed using Stata Statistical Software Release 12 (StataCorp., College Station, TX, USA).

### Ethical considerations

Ethical approval for the study was obtained from the Karolinska Institutet Regional Ethics Committee of Stockholm (recordals 2009/1985-31/4, 2011/1377-32, and 2012/2159-32). The committee approved that no written or verbal consent had to be given by the study subjects, as the study only pertained to extracting limited clinical data from patient charts. Patient records were anonymized and de-identified prior to analysis.

## Results

### Risk factors for infection

Patient characteristics of the *K*. *pneumoniae* and the *E*. *coli* cohort are presented in Table 1 (multivariable analysis) and S1 Table (univariate analysis). The median age was 68 years and 348/599 patients (58%) were male. In multivariable analysis, hematological, colorectal and bile/liver/pancreatic malignancies, OR 1.70 (95% CI 1.07–2.70), 2.56 (95% CI 1.34–4.89) and 3.45 (95% CI 1.77–6.75) respectively, were more common in the *K*. *pneumoniae* cohort, as well as Chronic Obstructive Pulmonary Disease (COPD), OR 1.96 (95% CI 1.14–3.36), kidney disease, OR 1.90 (95% CI 1.28–2.82), peripheral vascular disease, OR 3.74 (95% CI 1.65–8.48) and bile disease, OR 3.10 (1.44–6.66). Urinary catheters/abnormalities (including urinary ileostomy, suprapubic catheter and indwelling urinary catheter) and central venous catheters or ports were associated with *K*. *pneumoniae* infection, OR 2.36 (95% CI 1.64–3.40) and 2.32 (95% CI 1.53–3.54) respectively. *K*. *pneumoniae* were significantly more often healthcare-associated community-onset infections, OR 3.06 (95% CI 2.03–4.62). An identified urinary tract source of infection was more frequent for *E*. *coli*. Invasive infection caused by *K*. *pneumoniae* was significantly more often polymicrobial, even when excluding cases with growth of both *K*. *pneumoniae* and *E*. *coli*, OR 1.42 (95% CI 1.00–2.00).

**Table 1:** Clinical characteristics of patients with invasive infection caused by *K*. *pneumoniae* versus *E*. *coli*, multivariable analysis.

	*K*. *pneumoniae*	*E*. *coli*	Adjusted odds
	n = 599	*n = 599*	ratio
*Patients factors*	No (%)	*No (%)*	(95% CI)
			*K*. *pneumoniae* vs
			*E*. *coli*
Peripheral vascular disease	30 (5)	14 (2)	3.74 (1.65–8.48)
COPD	58 (10)	37 (6)	1.96 (1.14–3.36)
Kidney disease	105 (18)	69 (12)	1.90 (1.28–2.82)
Bile disease	36 (6)	15 (3)	3.10 (1.44–6.66)
Hematological malignancy	112 (19)	76 (13)	1.70 (1.07–2.70)
Bile/liver/pancreas malignancy	51 (9)	22 (4)	3.45 (1.77–6.75)
Colorectal malignancy	42 (7)	24 (4)	2.56 (1.34–4.89)
Urinary catheter	191 (32)	111 (19)	2.36 (1.64–3.40)
Central catheter	190 (32)	96 (16)	2.32 (1.53–3.54)
Hospital-acquired^a)^	178 (30)	197 (33)	0.53 (0.37–0.77)
Healthcare- associated community-onset^a)^	163 (27)	55 (9)	3.06 (2.03–4.62)

a) in relation to community-acquired infection

Additional factors (non-significant) included in the final multivariable analysis: arrhythmia, cerebrovascular disease with sequela, intestinal disease (ulcerative colitis, Crohn´s disease, op-ileostomy, intestinal co-infection), breast malignancy and melanoma

### Mortality

**Fig 1:**
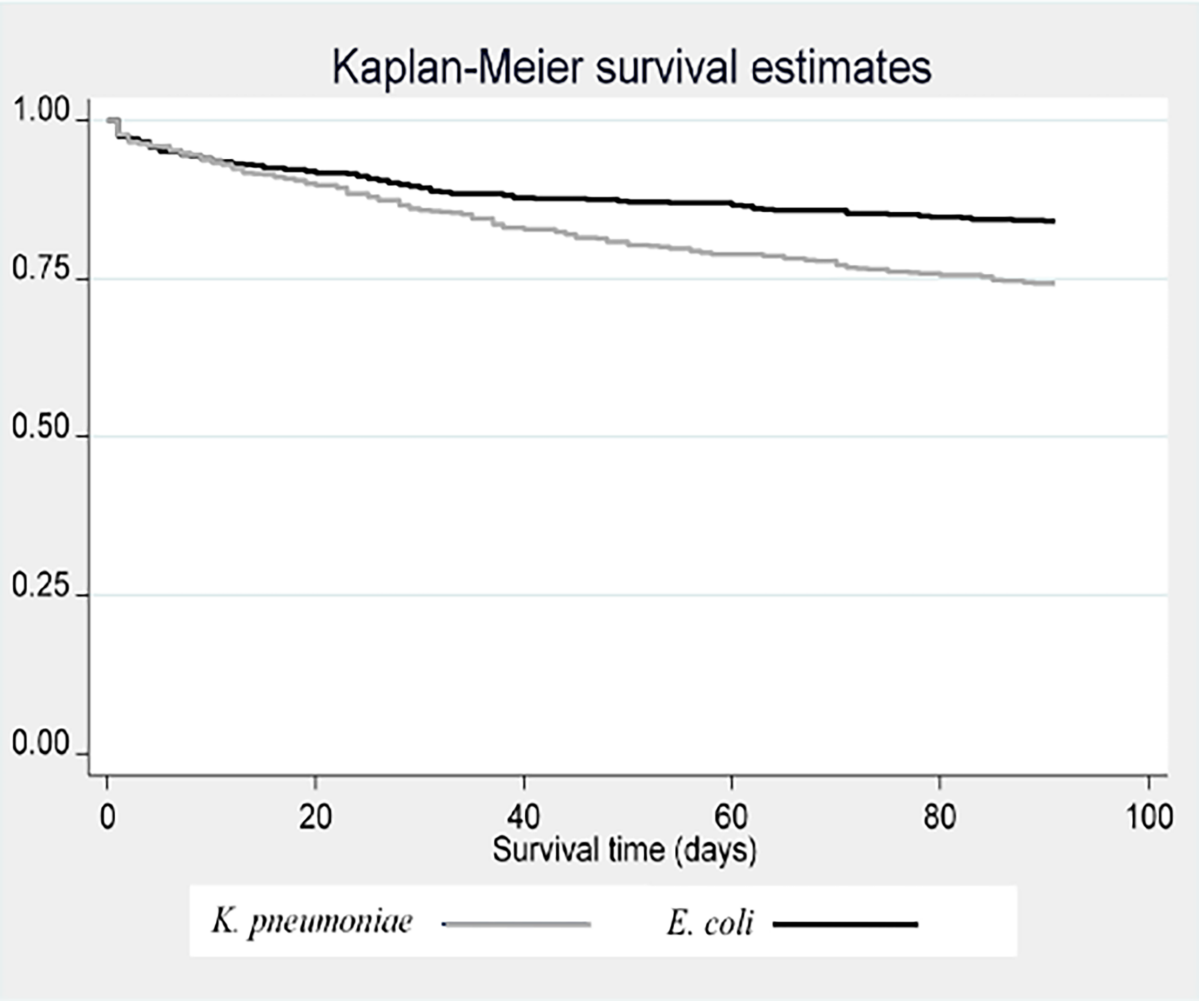
Kaplan-Meier survival curve *K*. *pneumoniae* versus *E*. *coli* cohort during 90 days. Log-rank test p<0.001.

The 7-day–and 30-day mortalities were similar in the *K*. *pneumoniae* and *E*. *coli* cohorts, 34/599 (6%) versus 39/599 (7%), p = 0.55, and 87/599 (15%) versus 70/599 (12%), p = 0.15 respectively. The 90-day mortality was significantly higher in the *K*. *pneumoniae* 156/599 (26%) versus 101/599 (17%) in the *E*. *coli* cohort, p<0.001. As seen in the Kaplan-Meier survival analysis (Fig 1) mortality difference increased between *K*. *pneumoniae* and *E*. *coli* over time. However, there was no increased 90-day mortality in patients with *K*. *pneumoniae* OR 1.17 (95% CI 0.85–1.62), after adjustment for polymicrobial infections, site of acquisition (healthcare-acquired, community-acquired, or healthcare-associated community-acquired), source of infection and comorbidities (S2 Table).

Predictors for mortality were evaluated in the extended *K*. *pneumoniae* cohort (n = 652). Associated factors significant in multivariable analysis are presented in Table 2, and univariate analyses are shown in S3 Table. High age, lung malignancy and infections emanating from the lungs were factors associated both with early (within 7 days) and late (up to 90 days) mortality. For mortality within 30 and 90 days host factors were of major importance. Patients with Charlson index >5, compared to patients with Charlson index ≤5, had an adjusted OR of 7.17 (95% CI 2.69–19.14) and 11.82 (95% CI 5.32–26.24) (not shown in table) respectively for fatal outcome within 30 and 90 days respectively. Within 30 and 90 days, mortality was also higher among patients with a hospital-acquired or a healthcare-associated infection, OR 1.99 (95% CI 1.21–3.28) and 1.69 (95% CI 1.02–2.79) respectively compared to community-acquired infection. Polymicrobial infection was a poor prognostic factor for mortality within 7 days, OR 3.07 (95% CI 1.51–6.27) (Table 2). Infections emanating from the lungs were associated with higher mortality than infections with other origins and were also more common among patients at the ICU.

**Table 2:** Associated factors for mortality in the extended *K*. *pneumoniae* cohort, factors significant in multivariable analysis.

	Mortality within	Mortality within	Mortality within
	7d	30 d	90 d
Associated factors*	(n = 43)	(n = 101)	(n = 176)
	Adjusted OR	Adjusted OR	Adjusted OR
Age	1.03 (1.00–1.05)	1.02 (1.00–1.04)	1.03 (1.01–1.04)
Polymicrobial infection	3.07 (1.51–6.27)	2.20 (1.32–3.68)	
Kidney disease			2.33 (1.41–3.84)
CNS disease		3.12 (1.73–5.62)	2.09 (1.26–3.45)
Lung malignancy	13.45 (3.94–45.90)	13.20 (4.11–42.38)	20.77 (6.01–71.73)
Urogenital, GI, bile/liver/pancreas malignancy		2.07 (1.10–3.91)	3.07 (1.87–5.05)
Hematological malignancy		3.13 (1.47–6.63)	2.50 (1.33–4.72)
Other malignancy**	6.18 (1.87–20.41)	6.34 (2.54–15.85)	3.77 (1.63–8.74)
Hospital-acquired			1.99 (1.21–3.28)
Healthcare-associated community-onset			1.69 (1.02–2.79)
*Source of infection*			
Respiratory tract	3.62 (1.01–13.04)	3.79 (1.32–10.87)	3.74 (1.44–9.68)
Bile/liver, GI		1.92 (1.00–4.16)	1.91 (1.15–3.15)
Unknown		2.09 (1.05–4.16)	

*Compared to variable being absent except for hospital-acquired and healthcare-associated community-onset where compared to community-acquired infection and for source of infection where compared to urinary tract.

**Breast, miscellaneous, melanoma

Variables included in models: 7-d mortality: age, polymicrobial infection, malignancies, onset of disease and source of infection. 30-d and 90-d mortality: age, polymicrobial infection, malignancies, onset of disease, source of infection, CNS disease, cardiovascular disease, lung disease and kidney disease.

The time to adequate antimicrobial treatment was almost equal in the two cohorts. Within 2 hours 210 (35%) versus 194 (32%) (p = 0.33) patients in the *K*. *pneumoniae*- and *E*. *coli* cohorts, within 4 hours 356 (59%) versus 373 (62%) (p = 0.31), and finally within 24 hours 525 (89%) versus 541 (91%) patients received adequate antimicrobial treatment. Out of the 68 patients in the *K*. *pneumoniae* cohort receiving adequate antibiotics after more than 24 h, or not receiving antibiotics at all, 20 (29%) died within 90 days (p = 0.47 compared to those receiving antibiotics within 24 hours).

### Bacterial characteristics and antimicrobial resistance

The isolates from Karolinska University Hospital showed low levels of resistance (S4 Table) in the *K*. *pneumoniae* cohort (n = 599). Only nine isolates (2%) were extended-spectrum beta-lactamase (ESBL)-producers, including one strain that also produced VIM-metallo-beta-lactamase (detected in a patient who had been hospitalized abroad). The most common resistance was against trimethoprim-sulfamethoxazole (n = 62, 10%) and ciprofloxacin (n = 47, 8%). A total of 106 isolates (18%) exhibited resistance to at least one antimicrobial group while only 15 (3%) showed multidrug-resistance, i.e. were resistant to ≥ three classes of antimicrobials. Among the *E*. *coli* isolates the susceptibility pattern was different showing resistance in 29% (n = 172) against trimethoprim-sulfamethoxazole, 17% (n = 103) against ciprofloxacin, and 6% (n = 36) were ESBL-producers, all p<0.001 compared to *K*. *pneumoniae*.

**Fig 2:**
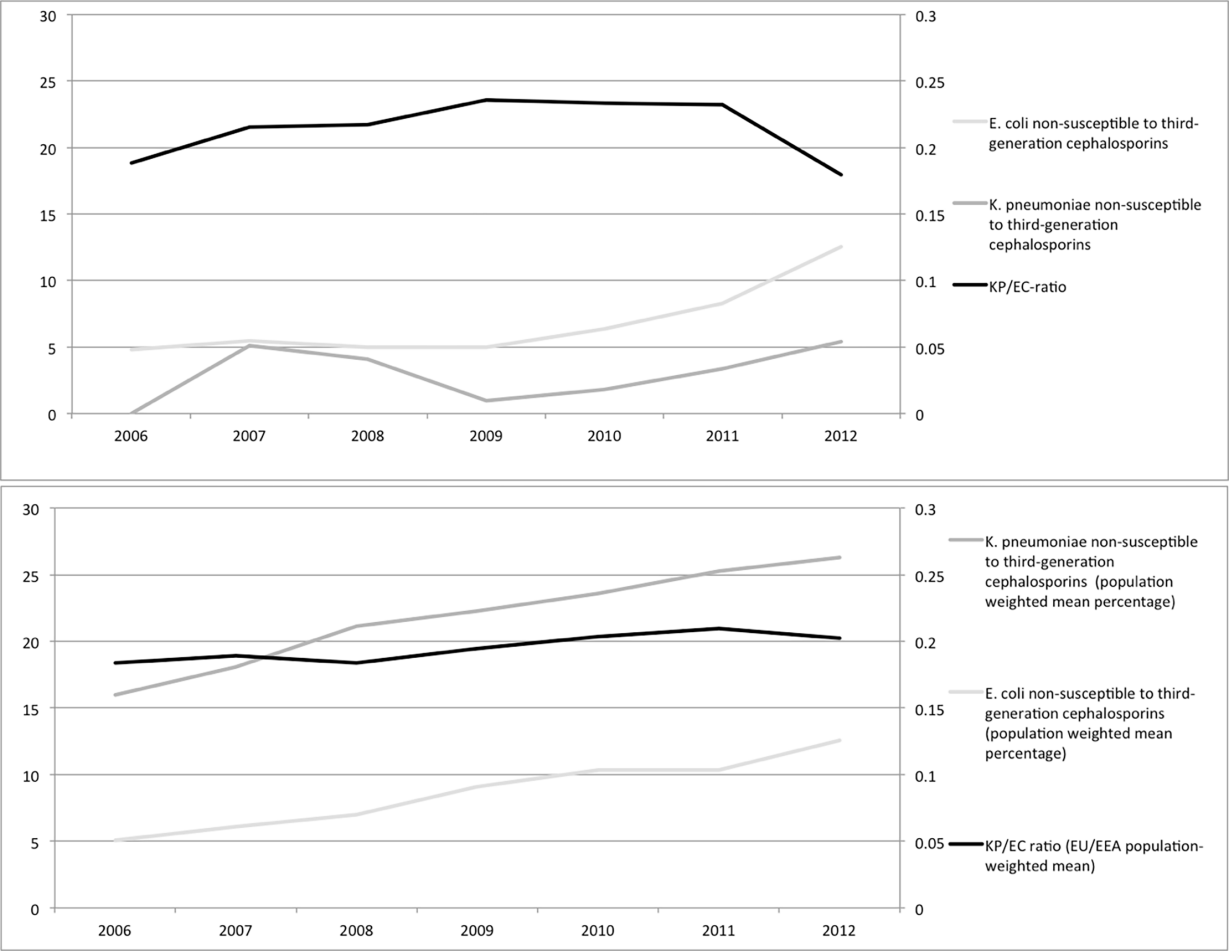
Rates of invasive isolates non-susceptible to third-generation cephalosporins among *K*. *pneumoniae* and *E*. *coli* 2006–2012, and *K*. *pneumoniae* / *E*. *coli* (KP/EC) ratio. A) Karolinska University Hospital. B) Twenty countries within EU/EEA reporting to EARS-Net. Population-weighted data.

In total (children and adults, reporting 1 patient/year) there were an increasing number of *K*. *pneumoniae* invasive infections from 2006 (70 patients) to 2012 (111 patients) (data not shown in table). A similar increase was observed of invasive *E*. *coli* infections from 356 in 2006 to 557 in 2012, so the proportion of *K*. *pneumoniae* to *E*. *coli* (KP/EC-ratio) ranged between 0.18–0.23 from year to year, but showed no changing trend during the period (p = 0.52). During this period the frequency of obtained blood cultures at the hospital has increased in total, but the ratio of positive cultures has remained unchanged (unpublished laboratory statistics, Karolinska University Hospital). Among the *K*. *pneumoniae* isolates there was no increase in the proportion of ESBL-producers during the period. In contrast, for *E*. *coli* ESBL-producing isolates increased significantly, from 2% 2006 to 11% 2012 (p<0.001). The proportion of non-susceptibility among third-generation cephalosporins (Fig 2A) in data from Karolinska University Hospital increased from 5 to 13% among the *E*. *coli* isolates and varied between 0 and 5% among the *K*. *pneumoniae* isolates.

### European data

**Fig 3:**
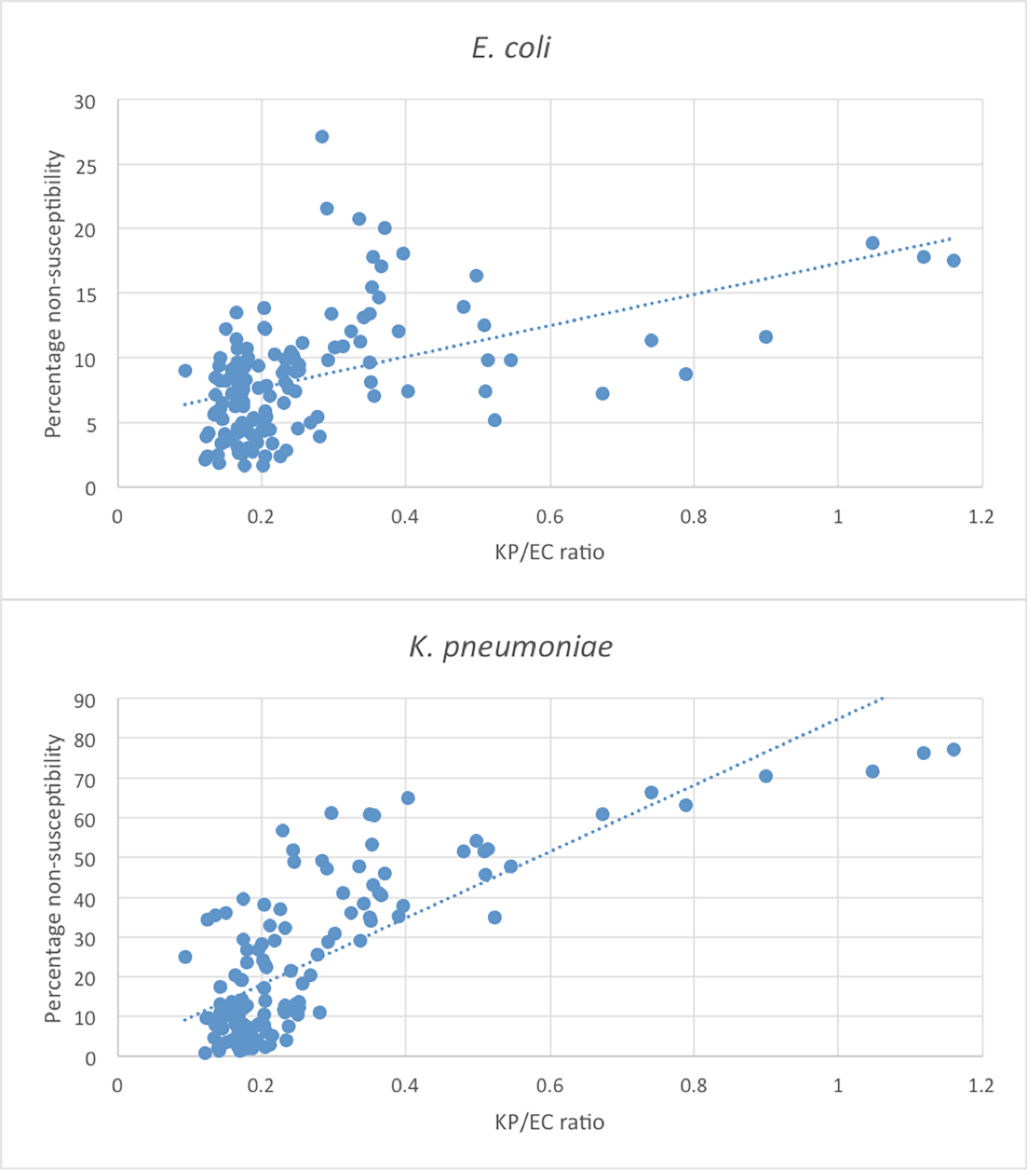
**The percentages of *E*. *coli* (3A) and *K*. *pneumoniae* (3B) isolates non-susceptible against third-generation cephalosporins plotted against the ratios of *K*. *pneumoniae/E*. *coli* (KP/EC ratio) among 20 European countries 2006–2012.** Each dot (n = 140) represents one country a certain year.

The EU/EEA population-weighted proportion of isolates non-susceptible to third-generation cephalosporins increased over time, both among *K*. *pneumoniae* (from 16% to 26% in the 20 countries) and in *E*. *coli* (from 5% to 13%). A moderate correlation between an increase in KP/EC-ratio and non-susceptibility to third-generation cephalosporin was observed for *E*. *coli* (r: 0.48 p<0.001) and the correlation was even more pronounced for non-susceptible *K*. *pneumoniae* (r: 0.77 p<0.001) (Fig 3). Also, the increasing third-generation cephalosporin-non-susceptibility in *E*. *coli* and *K*. *pneumoniae* paralleled a shift in the KP/EC-ratio 2006–2012 (p<0.001). In the Stockholm area, an increase in third-generation cephalosporin-resistant *E*. *coli* was observed, but contrary to the EARS-Net data, no change was observed in the KP/EC-ratio (p = 0.90) (Fig 2).

## Discussion

Invasive infection caused by *K*. *pneumoniae* is a disease with high 90-days mortality. There is a high level of comorbidity among the affected patients. Due to low resistance rates in *K*. *pneumoniae* in Sweden we could reduce the risk of bias that is often observed in other studies in assessment of mortality. In concordance with previous studies there are differences in patient populations affected by invasive infection caused by *K*. *pneumoniae* versus *E*. *coli* with a higher level of comorbidity in patients infected with *K*. *pneumoniae*. Patients with an impaired host defense are at greater risk of developing invasive infection caused by *K*. *pneumoniae* compared to *E*. *coli*, in this study 163/599 patients (27%) had a healthcare-associated community-onset infection in the *K*. *pneumoniae* cohort compared to only 55/599 (9%) in the *E*. *coli* cohort. In settings with a high prevalence of third-generation cephalosporin resistance among *K*. *pneumoniae*, the knowledge about risk factors for infections caused by this pathogen may have implications for selection of empiric treatment.

The *K*. *pneumoniae* isolates were in the Swedish setting more susceptible to antimicrobials than the *E*. *coli* isolates. This contrasts with most European countries where *K*. *pneumoniae* is associated with higher resistance levels compared to *E*. *coli*. The few countries reporting higher third-generation non-susceptibility in *E*. *coli* compared to *K*. *pneumoniae* all had low resistance frequencies, just as the cohort from Karolinska University Hospital [27]. As scatterplots in Fig 3A and 3B graphically show, there is a correlation between the ratio of *E*. *coli/K*. *pneumoniae* and the percentage of *E*. *coli* and *K*. *pneumoniae* isolates non-susceptible to third-generation cephalosporins. Among the isolates from the Swedish cohort, we could not see the same trend over time. There are reasons to believe that the ecological niches and resistance rates could be influenced by circulating epidemic clones [28–30]. The *K*. *pneumoniae*-isolates from patients admitted to Karolinska University Hospital Solna 2007–2009 (n = 139) were in a previous study subjected to molecular analysis [31]. In that study, there was a high level of diversity among sequence types, and internationally reported epidemic high-risk clones were not detected. The low antimicrobial resistance among *K*. *pneumoniae* isolates in the present study supports the previous results that there is no indication of circulating high-risk clones in the Stockholm area, which could offer an explanation to the relatively low proportion of *K*. *pneumoniae* in blood cultures. The 30-d mortality of 16% noted in our study is compared to other studies low, but comparison is not easily made between e.g. 30-d mortality and in-hospital mortality, and between settings of high-and low prevalence of ESBL-producers. Since most patients affected by invasive infection caused by *K*. *pneumoniae* have a known malignancy, studies investigating if invasive infection caused by *K*. *pneumoniae* should be regarded as a predictor for an underlying undiagnosed malignancy, would be of interest.

Due to the retrospective design of our study we could not control for all possible variables and data from the medical records can be incomplete. We lacked the possibility to retrieve severity score indexes (i.e. disease severity on onset of illness), a factor influencing both time to antimicrobial treatment and mortality. However, the study is large, containing nearly 600 patients in each group. As the *E*. *coli* cohort was selected to match the *K*. *pneumoniae* cohort in sex and age, results from this study are not generalizable to all *E*. *coli* invasive infections. Data from EARS-Net are much less detailed compared to the Karolinska University Hospital data, but the similar sampling framework (restricting data to invasive isolates and using the same algorithm for avoiding isolate duplication) allowed for a crude overall comparison without any patient-specific stratifications. It should however be noted that the pooled European data are based on various national surveillance systems with significant differences in population coverage and BSI case ascertainment of patients with BSIs. In countries where patients with community-acquired infections less frequently are sampled compared to those with healthcare-associated and hospital-acquired infections, the KP/EC-ratio might be overestimated. We believe that excluding the countries reporting <300 isolates of *E*. *coli* per year minimized this potential bias.

In summary, invasive infections caused by *K*. *pneumoniae* affect patients with high comorbidity. The disease is associated with high long-term mortality, even in a low-prevalence setting of ESBL-producers. Specific risk factors for invasive infection caused by *K*. *pneumoniae* compared to *E*. *coli* are healthcare-associated community-onset infection, malignancies, peripheral vascular disease, COPD, kidney disease, bile disease, and indwelling catheters. In settings with high resistance among *K*. *pneumoniae* this information could be taken into consideration when choosing empiric antibiotic treatment in severely ill patients. The increasing KP/EC-ratio observed in European data was not demonstrated in the Karolinska University Hospital data, and could be due to absence of epidemic *K*. *pneumoniae* clones, as the latter could explain both high prevalence of resistance and a higher overall burden of *K*. *pneumoniae* BSI.

## Supporting information

S1 Fig Inclusion chart. (TIFF)

S1 Table Clinical characteristics of patients with invasive infection caused by *K*. *pneumoniae* versus *E*. *coli*, univariate analysis. (PDF)

S2 Table Mortality within 90 days *K*. *pneumoniae* in comparison with *E*. *coli*, multivariable analysis. (PDF)

S3 Table Associated factors for mortality within 7, 30 and 90 days in the extended *K*. *pneumoniae* cohort (n = 652). (PDF)

S4 Table Antibiotic resistance in the *K*. *pneumoniae* versus *E*. *coli* cohort. No of isolates with non-susceptibility (intermediate and resistant) to antimicrobials.
(PDF)
