# Supplementary figures and images for: Invasive infection caused by Klebsiella pneumoniae is a disease affecting patients with high comorbidity and associated with high long-term mortality
Source: PLoS One. 2018 Apr 6;13(4):e0195258. doi: 10.1371/journal.pone.0195258 (PMC5889183; doi:10.1371/journal.pone.0195258)

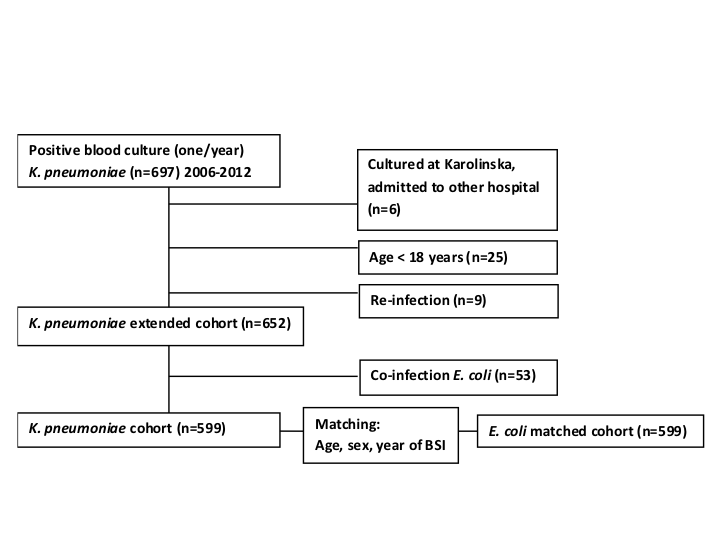

Supplement: S1 Fig — (TIFF) [file pone.0195258.s001.tiff]
