# Supplementary material for: Invasive infection caused by Klebsiella pneumoniae is a disease affecting patients with high comorbidity and associated with high long-term mortality
Source: PLoS One. 2018 Apr 6;13(4):e0195258. doi: 10.1371/journal.pone.0195258 (PMC5889183; doi:10.1371/journal.pone.0195258)
Supplement: S1 Table — (PDF) [file pone.0195258.s002.pdf]

Patients factors	***K. pneumoniae*** **n=599 No (%)**	***E. coli*** **n=599 No (%)**	**p-value univariate analysis**	**Odds ratio (95% confidence interval)**
Age, median, years	68	68		N/A
Male sex	348 (58)	348 (58)		N/A
Charlson index, median	3	2	**<0.001**	N/A
Charlson index 0-1	85 (14)	206 (34)	N/A	1 (ref)
Charlson index 2-3	288 (48)	247 (41)	**<0.001**	**3.05 (2.19-4.25)**
Charlson index 4-5	116 (19)	70 (11)	**<0.001**	**4.18 (2.78-6.32)**
Charlson index >5	110 (18)	76 (12)	**<0.001**	**3.82 (2.51-5.81)**
Diabetes [a)]	116 (19)	99 (17)	0.21	1.21 (0.90-1.62)
Heart disease [b)]	118 (20)	113 (19)	0.70	1.06 (0.78-1.45)
Previous AMI	41 (7)	40 (7)	0.91	1.03 (0.65-1.63)
Angina pectoris	30 (5)	31 (5)	0.90	0.97 (0.58-1.62)
Congestive heart failure	55 (9)	58 (10)	0.76	0.94 (0.63-1.40)
Arrhythmia	55 (9)	58 (10)	0.05	0.69 (0.47-1.00)
Peripheral vascular disease	30 (5)	14 (2)	**0.01**	**2.33 (1.19-4.59)**
Hypertension	123 (21)	144 (24)	0.14	0.81 (0.62-1.07)
Lung disease [c)]	112 (19)	93 (16)	0.28	1.03 (0.98-1.09)
COPD	58 (10)	37 (6)	**0.03**	**1.64 (1.05-2.57)**
Asthma	20 (3)	16 (3)	0.51	1.25 (0.65-2.41)
Pleural effusion	3 (1)	6 (1)	0.33	0.50 (0.13-2.00)
Kidney disease [d)]	105 (18)	69 (12)	**0.003**	**1.64 (1.18-2.29)**
Decreased kidney function (moderate to severe)	83 (14)	67 (11)	0.17	1.28 (0.90-1.80)
Polycystic kidneys	11 (2)	8 (1)	0.49	1.38 (0.55-3.42)
Diabetic nephropathy	9 (2)	10 (2)	0.82	0.90 (0.37-2.21)
Liver disease (mild)	9 (2)	11 (2)	0.66	0.82 (0.34-1.97)
Liver disease (moderate-severe)	43 (7)	29 (5)	0.07	1.64 (0.96-2.78)
Bile disease	36 (6)	15 (3)	**0.003**	**2.75 (1.42-5.32)**
CNS-disease [e)]	129 (22)	118 (120)	0.42	1.13 (0.84-1.50)
Cerebrovascular disease, no sequela	30 (5)	30 (5)	1.00	1.00 (0.59-1.69)
Cerebrovascular disease, sequela	45 (9)	29 (5)	0.06	1.57 (0.98-2.52)
Parkinson	5 (1)	8 (1)	0.37	0.57 (0.17-1.95)
MS	6 (1)	6 (1)	1.00	1.00 (0.32-3.10)
Dementia	34 (6)	32 (5)	0.80	1.07 (0.64-1.77)
Epilepsy	5 (1)	9 (2)	0.29	0.56 (0.19-1.66)
Other CNS-disease [f)]	18 (3)	25 (4)	0.29	0.72 (0.39-1.32)

Crohn´s disease/ulcerative colitis	15 (3)	10 (2)	0.32	1.50 (0.67-3.34)
Diverticulosis/-itis	12 (2)	14 (2)	0.67	0.83 (0.36-1.93)
GVH-reaction intestine	8 (1)	0 (0)	**0.01****	N/A
Intestinal disease [g)]	65 (11)	47 (8)	0.06	1.49 (0.98-2.26)
Rheumatic disease [h)]	12 (2)	17 (3)	0.92	1.02 (0.70-1.48)
Gastric ulcer	5 (1)	7 (1)	0.57	0.71 (0.23-2.25)
Malignancy, all*	318 (53)	226 (38)	**<0.001**	**2.07 (1.60-2.67)**
Hematological	112 (19)	76 (13)	**0.005**	**1.58 (1.15-2.17)**
Urogenital	76 (13)	67 (11)	0.38	1.19 (0.81-1.76)
Colorectal	42 (7)	24 (4)	**0.013**	**1.95 (1.15-3.30)**
Pulmonary	15 (3)	19 (3)	0.48	0.78 (0.39-1.56)
Bile/liver/pancreas	51 (9)	22 (4)	**<0.001**	**2.71 (1.55-4.72)**
Breast	12 (2)	5 (1)	0.10	2.40 (0.85-6.81)
Ventricle/esophagus	2 (0)	5 (1)	0.27	0.40 (0.0-2.06)
Melanoma	5 (1)	0 (0)	**0.007****	N/A
Miscellaneous	12 (2)	15 (3)	0.70	0.86 (0.40-1.85)
Metastasized	92 (15)	65 (11)	**0.02**	**1.55 (1.08-2.22)**
Previous organ transplant[i)]	51 (9)	46 (8)	0.43	1.19 (0.77-1.86)
Neutropenia	81 (14)	64 (11)	0.12 [a)]	1.33 (0.93-1.92)
Community-acquired infection	258 (43)	347 (58)	**<0.001**	**0.55 (0.44-0.70)**
Healthcare-associated infection	163 (27)	55 (9)	**<0.001**	**3.57 (2.54-5.03)**
Hospital-acquired infection	178 (30)	197 (33)	0.24	0.87 (0.68-1.10)
Urinary catheter [j)]	191 (32)	111 (19)	**<0.001**	**2.19 (1.64-2.93)**
Central catheter [k)]	190 (32)	96 (16)	**<0.001**	**2.47 (1.85-3.30)**
Ileostomy	28 (5)	13 (2)	**0.02**	**2.25 (1.14-4.44)**
Surgery within 30 d	76 (13)	81 (14)	0.65	0.92 (0.65-1.31)
Dialysis (HD, PD)	22 (4)	10 (2)	**0.033**	**2.33 (1.07-5.09)**
Hemodialysis	20 (3)	9 (2)	**0.040**	**2.38 (1.04-5.43)**
At ICU when onset of sepsis	24 (4)	6 (1)	**0.002**	**4.00 (1.64-9.79)**
Source of infection				
Urinary tract	236 (39)	385 (64)	**<0.001**	**0.35 (0.27-0.45)**
Respiratory tract	25 (4)	17 (3)	0.20	1.53 (0.80-2.94)
Bile/liver	89 (15)	46 (8)	**<0.001**	**2.23 (1.50-3.32)**
Gastrointestinal tract	57 (10)	52 (9)	0.62	1.10 (0.75-1.63)
CNS	11 (2)	0 (0)	**0.003****	N/A
Miscellaneous	16 (3)	8 (1)	0.11	2.00 (0.86-4.67)
Site unknown	165 (28)	92 (15)	**<0.001**	**2.14 (1.59-2.87)**
Polymicrobial infection	89 (15)	66 (11)	**0.047**	**1.42 (1.00-2.00)**
E. coli	-	-		

Viridans streptococci	11 (2)	13 (2)	0.67	0.83 (0.36-1.93)
S. aureus	11 (2)	7 (1)	0.35	1.57 (0.61-4.05)
Enterococcus faecium	8 (1)	7 (1)	1.00	1.00 (0.35-2.85)
Enterococcus faecalis	12 (2)	9 (2)	0.49	1.38 (0.55-3.42)

Bold = P<0.05

*sum will be exceeded when summing individual malignancies due to some patients having more than one type of cancer

**using McNemar test

[a)]insulin and/or tablet treated

[b)]previous AMI, aortic aneurysm >6 cm, congestive heart failure

[c)] COPD, asthma, cancer, pleural effusion

[d)]tumor, hydronephrosis, decreased function

[e)]cerebrovascular disease, dementia

[f)]polio, myasthenia gravis, lymphoma, previous brain surgery

[g)]ulcerative colitis, Crohn´s disease, op-ileostomy, intestinal co-infection

[h)] RA, SLE, vasculitis

[i)]kidney, liver, bone marrow, heart

[j)]indwelling urinary catheter, suprapubic catheter, bricker bladder, urinary ileostomy. Intermittent catheterization not included

[k)]central venous catheter, central venous port, intrathecal catheter
