# Supplementary material for: Invasive infection caused by Klebsiella pneumoniae is a disease affecting patients with high comorbidity and associated with high long-term mortality
Source: PLoS One. 2018 Apr 6;13(4):e0195258. doi: 10.1371/journal.pone.0195258 (PMC5889183; doi:10.1371/journal.pone.0195258)
Supplement: S2 Table — (PDF) [file pone.0195258.s003.pdf]

	Mortality within 90 days Odds Ratio (95% CI)
K. pneumoniae versus *E. coli*	1.17 (0.85-1.62)
Age	**1.03 (1.01-1.04)**
Sex	0.94 (0.68-1.30)
Polymicrobial infection	**1.55 (1.03-2.34)**
Cardiovascular disease	1.27 (0.86-1.88)
Lung disease	**2.02 (1.38-2.97)**
Kidney disease	**2.05 (1.34-3.16)**
CNS disease	**2.66 (1.81-3.91)**
Malignancy	**3.18 (2.22-4.53)**
Source of infection	
Urinary tract	1.0 (ref)
Respiratory tract[a)]	**6.15 (2.94-12.87)**
Gastrointestinal, bile/liver/pancreas, CNS [a)]	**2.84 (1.90-4.24)**
Source unknown [a)]	**2.53 (1.66-3.87)**
Community-acquired infection	1.0 (ref)
Hospital-acquired infection [b)]	**1.65 (1.13-2.42)**
Healthcare-associated community-onset [b)]	1.52 (1.00-2.31)

Bold = P<0.05

[a)]in relation to source of infection in the urinary tract

[b)]in relation to community-acquired infection
