# Supplementary material for: Invasive infection caused by Klebsiella pneumoniae is a disease affecting patients with high comorbidity and associated with high long-term mortality
Source: PLoS One. 2018 Apr 6;13(4):e0195258. doi: 10.1371/journal.pone.0195258 (PMC5889183; doi:10.1371/journal.pone.0195258)
Supplement: S3 Table — (PDF) [file pone.0195258.s004.pdf]

Clinical characteristics	**Day 0-7**			**Day 0-30**			**Day 0-90**		
	**Dead (n=43)**	**Survivor (n=609)**	**p-value**	**Dead (n=101)**	**Survivor (n=551)**	**p-value**	**Dead (n=176)**	**Survivor (n=476)**	**p-value**
Median age, years	72	66	0.15	71	67	**0.04**	70	67	**0.010**
Male sex	26 (60)	349 (57)	0.69	52 (51)	323 (59)	0.18	93 (53)	282 (59)	0.14
Charlson index 0-1	4 (9)	91 (15)	0.31	6 (6)	89 (16)	0.007	10 (6)	85 (18)	**<0.001**
Charlson index 2-3	20 (47)	292 (63)	0.86	45 (45)	267 (48)	0.47	72 (41)	240 (57)	**0.03**
Charlson index 4-5	6 (14)	118 (19)	0.38	15 (15)	109 (20)	0.25	27 (15)	97 (20)	0.15
Charlson index >5	13 (30)	108 (18)	**0.04**	35 (35)	86 (16)	**<0.001**	67 (38)	54 (11)	**<0.001**
Diabetes [a)]	6 (14)	119 (20)	0.37	14 (14)	111 (20)	0.14	28 (16)	97 (20)	0.20
Heart disease [b)]	11 (26)	121 (20)	**0.03**	28 (28)	104 (19)	**0.047**	45 (26)	87 (18)	**0.007**
Peripheral vascular disease	3 (7)	27 (4)	0.44	4 (4)	26 (5)	1.00	7 (4)	23 (5)	0.64
Lung disease [c)]	15 (35)	110 (18)	**0.007**	32 (32)	93 (17)	**0.001**	46 (26)	79 (17)	**0.006**
Kidney disease [d)]	7 (16)	103 (17)	0.92	17 (17)	93 (17)	0.99	37 (21)	73 (15)	0.09
Liver disease (mild)	1 (2)	8 (1)	0.46	2 (2)	7 (1)	0.64	4 (2)	5 (1)	0.26
Liver disease (moderate-severe)	4 (9)	42 (7)	0.53	9 (9)	37 (7)	0.43	14 (8)	32 (7)	0.59
Bile disease	0 (0)	40 (7)	1.00	2 (2)	38 (7)	0.06	8 (5)	32 (7)	0.30
CNS-disease [e)]	10 (23)	131 (22)	0.79	32 (32)	109 (20)	**0.008**	43 (24)	98 (21)	0.29
Intestinal disease [f)]	1 (2)	69 (11)	0.07	8 (8)	62 (11)	0.32	14 (8)	56 (12)	0.16
Malignancy, all	29 (67)	320 (53)	0.06	68 (67)	281 (51)	**0.002**	120 (68)	229 (48)	**<0.001**
Hematological	11 (26)	114 (19)	0.27	24 (24)	101 (18)	0.20	35 (20)	90 (19)	0.78
Lung	6 (14)	10 (2)	**<0.001**	8 (8)	8 (1)	**0.001**	12 (7)	4 (1)	**<0.001**
Urogenital	3 (7)	75 (12)	0.30	7 (7)	71 (13)	0.09	20 (11)	58 (12)	0.77
Ventricle/esophagus	0 (0)	3 (0)	1.00	1 (1)	2 (0)	0.40	2 (1)	1 (0)	0.18
Bile/liver/pancreas	4 (9)	54 (9)	0.79	13 (13)	45 (8)	0.13	26 (15)	32 (7)	**0.001**
Colorectal	0 (0)	45 (7)	0.06	4 (4)	41 (7)	0.21	13 (7)	32 (7)	0.77
Miscellaneous[g)]	5 (12)	28 (5)	**0.04**	12 (12)	21 (4)	**<0.001**	16 (9)	17 (4)	**0.004**
Metastasized	11 (26)	92 (15)	0.07	29 (29)	74 (13)	**<0.001**	57 (32)	46 (10)	**<0.001**
Previous organ transplant [h)]	2 (5)	54 (9)	0.57	5 (5)	51 (9)	0.16	12 (7)	44 (9)	0.33
Neutropenia	9 (21)	87 (14)	0.24	13 (13)	83 (15)	0.57	24 (14)	72 (15)	0.63
Community-acquired infection	19 (44)	267 (44)	0.97	36 (36)	250 (45)	0.07	60 (34)	226 (47)	**0.002**
Healthcare-associated community-onset	8 (19)	165 (27)	0.22	28 (28)	145 (26)	0.77	54 (31)	119 (25)	0.15

Hospital-acquired infection	16 (37)	177 (29)	0.26	37 (37)	156 (28)	0.09	62 (35)	131 (28)	0.06
Urinary catheter[i]	14 (33)	177 (29)	0.23	35 (35)	156 (28)	0.07	56 (32)	135 (28)	0.21
Central catheter [j]	16 (37)	190 (31)	0.41	38 (38)	168 (30)	0.16	63 (36)	143 (30)	0.16
In respirator	2 (5)	4 (1)	0.05	3 (3)	3 (1)	0.05	4 (2)	2 (0)	**0.05**
Other catheters/stomia	2 (5)	51 (8)	0.76	8 (8)	45 (8)	0.89	12 (7)	41 (9)	0.57
Surgery within 30 d	4 (9)	73 (12)	0.60	9 (9)	68 (12)	0.33	23 (13)	54 (11)	0.55
Dialysis at onset of BSI	2 (5)	21 (3)	0.66	6 (6)	17 (3)	0.15	9 (5)	14 (3)	0.18
Dialysis due to BSI	2 (5)	8 (1)	0.14	5 (5)	5 (1)	**0.01**	7 (4)	3 (1)	**0.005**
Polymicrobial	18 (42)	124 (20)	**0.001**	36 (36)	106 (19)	**<0.001**	50 (28)	92 (19)	**0.01**
Source of infection									
Urinary tract	12 (28)	233 (38)	0.18	24 (24)	221 (40)	**0.002**	48 (27)	197 (41)	**0.001**
Respiratory tract	5 (12)	21 (3)	**0.02**	9 (9)	17 (3)	**0.01**	13 (7)	13 (3)	**0.007**
Bile/liver	3 (7)	103 (17)	0.09	16 (16)	90 (16)	0.90	31 (18)	75 (16)	0.57
Abdominal	5 (12)	60 (10)	0.61	14 (14)	51 (9)	0.16	25 (14)	40 (8)	**0.03**
Miscellaneous	1 (2)	15 (2)	1.00	2 (2)	14 (3)	1.00	5 (3)	11 (2)	0.78
CNS	0 (0)	11 (2)	1.00	1 (1)	10 (2)	1.00	2 (1)	9 (2)	0.74
Site unknown	17 (40)	166 (27)	0.08	35 (35)	148 (27)	0.11	52 (30)	131 (28)	0.61
At ICU at time of sepsis	5 (12)	20 (3)	**0.02**	11 (11)	14 (3)	**<0.001**	14 (8)	11 (2)	**0.001**
To ICU due to sepsis	13 (30)	54 (9)	**<0.001**	18 (18)	49 (9)	**0.007**	24 (14)	43 (9)	0.09
*Time to antibiotic treatment**									
Treatment within 1 h	10 (24)	104 (17)	0.28	18 (18)	96 (18)	0.89	34 (20)	80 (17)	0.43
Treatment within 2 h	19 (45)	214 (35)	0.20	39 (39)	194 (36)	0.46	64 (37)	169 (36)	0.78
Treatment within 4 h	32 (76)	360 (60)	**0.03**	69 (70)	323 (59)	**0.05**	113 (65)	279 (60)	0.15
Treatment within 12 h	36 (86)	491 (81)	0.49	84 (85)	443 (81)	0.38	137 (79)	390 (83)	0.32
Treatment within 24 h	38 (90)	535 (89)	0.73	89 (90)	484 (89)	0.72	150 (87)	423 (90)	0.30
Treatment within 48 h	39 (93)	589 (98)	0.06	95 (94)	533 (98)	0.34	166 (96)	462 (98)	0.18
More than 48 h **	3 (7)	14 (2)	0.09	4 (4)	13 (2)	0.31	7 (4)	10 (2)	0.18
Bacterial characteristics									
ESBL	2 (5)	9 (2)	0.16	2 (2)	9 (2)	0.68	2 (4)	9 (2)	0.74

Bold = P<0.05

*calculations based on 645 patients due to missing data in 7 patients

**including patients never receiving antibiotics

[a)]insulin and/or tablet treated

[b)]previous AMI, aortic aneurysm >6 cm, congestive heart failure

[c)] COPD, asthma, cancer, pleural effusion, fibrosis

[d)]tumor, hydronephrosis, decreased function

[e]cerebrovascular disease, dementia

[f]ulcerative colitis, Crohn´s disease, op-ileostomy, intestinal co-infection

[g]breast, head and neck, skin cancer

[h]kidney, liver, bone marrow, heart

[i]indwelling urinary catheter, suprapubic catheter, bricker bladder, urinary ileostomy. Intermittent catheterization not included

[j]central venous catheter, central venous port, intrathecal catheter
