# Supplementary material for: Invasive infection caused by Klebsiella pneumoniae is a disease affecting patients with high comorbidity and associated with high long-term mortality
Source: PLoS One. 2018 Apr 6;13(4):e0195258. doi: 10.1371/journal.pone.0195258 (PMC5889183; doi:10.1371/journal.pone.0195258)
Supplement: S4 Table — No of isolates with non-susceptibility (intermediate and resistant) to antimicrobials. (PDF) [file pone.0195258.s005.pdf]

**Antibiotic**	***K. pneumoniae*** **(n=599) (%)**	***E. coli*** **(n=599) (%)**	***K. pneumoniae*** **vs.** ***E. coli*** **P-value**	***E. coli*** **(n=3047) ***	**P-value** ***E. coli*** **matched cohort versus all** ***E. coli***
Ciprofloxacin	47 (8)	103 (17)	**<0.001**	593 (19)	0.20
Trimethoprim/ sulfa	62 (10)	172 (29)	**<0.001**	898 (29)	0.73
Cefotaxime	11 (2)	40 (7)	**<0.001**	208 (7)	0.90
Ceftazidime	15 (3)	35 (6)	**0.004**	193 (6)	0.65
Gentamicin	13 (2)	40 (7)	**<0.001**	194 (6)	0.63
Piperacillin/ tazobactam	35 (6)	43 (7)	0.35	298 (10)	**0.046**
Meropenem	2 (0)	0 (0)	0.50	0 (0)	1.00
Multidrug-resistance (I or R ≥3 classes of antibiotics)	15 (3)	57 (10)	**<0.001**	209 (7)	**0.022**
ESBL (including CPE)	9 (2)	36 (6)	**<0.001**	172 (6)	0.72
CPE**	1 (0)	0 (0)	1.00	0 (0)	1.00
Isolates with resistance to any group above	106 (18)	218 (36)	**<0.001**	1226 (40)	0.08

Bold = P<0.05

* includes children and one isolate/patient/year

** VIM, CTX-M-1, CMY-2 in genotypic analysis
